# The Persistence of Neuromyths in the Educational Settings: A Systematic Review

**DOI:** 10.3389/fpsyg.2020.591923

**Published:** 2021-01-12

**Authors:** Marta Torrijos-Muelas, Sixto González-Víllora, Ana Rosa Bodoque-Osma

**Affiliations:** ^1^Psychology Department, Faculty of Education of Cuenca, University of Castilla-La Mancha, Cuenca, Spain; ^2^Department of Physical Education, Arts and Music, Faculty of Education of Cuenca, University of Castilla-La Mancha, Cuenca, Spain

**Keywords:** neuromyths, teachers, pre-service teachers, educators, neuroeducation, neuroscience

## Abstract

Neuroscience influences education, and these two areas have converged in a new field denominated “Neuroeducation.” However, the growing interest in the education–brain relationship does not match the proper use of research findings. In 2007, the Organization for Economic Cooperation and Development (OECD) warned of the misunderstandings about the brain among teachers, labeling them as neuromyths. The main objective here is to observe the prevalence of the neuromyths in educators over time. After two decades of publications of research on neuromyths among in-service or prospective teachers, this work presents a systematic scientific review. To select the articles, we used the words: “teachers,” “preservice teachers,” “neuromyths” combined with the Boolean data type “and.” The search was filtered according to the following criteria: (a) identifiable author, (b) written in English, Spanish, French, Italian, or Portuguese, (c) word neuromyth in title, abstract, or keywords, (d) research with a participant's survey, (e) sample focused on educators, (f) peer-review publication index in JCR, SJR, or ESCI. The documents were found through Web of Science, Scopus, PubMed, Dialnet, ProQuest, EBSCO-host, and Google Scholar. After the search, 24 articles were identified as being of sufficiently high quality for this systematic review. This result highlights that neuromyths are still the subject of attention almost two decades after their definition. The findings present neuromyths as the consequence of a lack of scientific knowledge, a communicative gap between scientists and teachers, and the low-quality information sources consulted by teachers. In addition, the data on protectors and predictors of neuromyths is inconsistent. There is also no standard scientific methodology nor a guideline to determine a new neuromyth. The results show the need to improve the scientific content in higher education and the importance of in-service teacher training. This research justifies the requirement for university professors to be active researchers and to establish a close link with educators from other fields and levels. Neuroeducation will be the bridge that unites scientific knowledge and practical application in education, with a rigorous, standard method for the entire scientific-educational community.

## Introduction

### A Brief History of the Neuromyths

Neuromyth is not a new concept. The word was first coined during the 1980s when the neurosurgeon Alan Crockard used it to describe a misleading concept about the brain function in the discipline of medicine (Howard-Jones, 2014; Fuentes and Risso, 2015). From an educational approach, a neuromyth was described as “a misconception generated by a misunderstanding, a misreading, or a misquoting of facts scientifically established (by brain research) to make a case for the use of brain research in education and other contexts” (OECD, 2002). Since that definition appeared, previous studies have emphasized the widespread presence of the neuromyths and their persistence, especially among individuals in contact with education (Howard-Jones et al., 2009; Dekker et al., 2012; Howard-Jones, 2014; Ferrero et al., 2016; Düvel et al., 2017; among others).

In 2002, the UK's OECD launched the Brain and Learning project (Howard-Jones, 2014), and Herculano-Houzel (2002) published the first survey about knowledge of the brain. She included 95 multiple-choice assertions, 83 related to the information that the general public has about brain research (Herculano-Houzel, 2002) and several neuromyths.

Five years later, the OECD wrote about the proliferation of the neuromyths around (a) critical periods, (b) the age of three as the time when everything important is decided, (c) multilingualism, (d) left vs. right brain people, and (e) the 10% of the use of our brain, as the most widely spread neuromyths. Most neuromyths are built in the base of a kernel of truth (Grospietsch and Mayer, 2018, 2019), i.e., valid scientific findings support them (Dekker et al., 2012), but they were adulterated because of misinterpretations, oversimplifications (Howard-Jones, 2014), and even due to a flawed interpretation of scientific results (Pasquinelli, 2012; Howard-Jones, 2014).

Research has provided evidence against neuromyths. As an example, neuroimaging research has demonstrated that both hemispheres are responsible for most of the procedures and are in constant communication, even though they differ in their functions (Ansari, 2008), which runs counter to myths such as left vs. right brain people, or multiple intelligences (Geake, 2008).

The myth about only 10% of brain use seems to be the most enduring neuromyth. It has survived more than a century. In 1907, Williams James wrote about the idea that humans used mental and physical resources below their means (James, 1907). Later, physicist Albert Einstein in a radio interview in 1920 (Pallarés-Domínguez, 2016), encouraged people to think more (Geake, 2008; Dündar and Gündüz, 2016; Papadatou-Pastou et al., 2017). He invited people to enhance their possibilities, using more than 10% of their brain, but he did not intend to spread such a colossal misunderstanding. However, as reported in the previous literature, not only can excellent scientific data be behind a neuromyth but also a neuroanatomical fact. The glia-neuron rate (or white matter-gray matter) which is one for every ten (Pasquinelli, 2012) may be responsible for the myth that claims that humans only use the 10% of their brain (because of the aforementioned rates). Scientific research shows how improbable this assertion may be, just taking into consideration that no one single brain area is 100% “out of work,” even when sleeping (Centre for Educational Research and Innovation and OECD, 2007).

Closely related to education, we can find the neuromyth of the visual, auditory, and kinaesthetic (VAK) learning styles. Under this approach, every child has a dominant learning style, which should be identified to teach each of them more precisely and create lesson plans according to their preferences (Geake, 2008; Macdonald et al., 2017). In this case, the kernel of truth is found in an oversimplification (Ansari, 2008) of fundamental research that has identified different parts of the brain that process visual, auditory, or kinaesthetic information (Dekker et al., 2012), i.e., different regions of the cortex have specific roles in sensory processing (Howard-Jones, 2014). Lack of evidence in VAK/learning styles has been successfully established (Pashler et al., 2008; Riener and Willingham, 2010; Willingham et al., 2015). Nevertheless, it is one of the most deeply rooted and widely believed neuromyths (Rodrigues Rato et al., 2013; Deligiannidi and Howard-Jones, 2015; Papadatou-Pastou et al., 2017, 2018; Varas-Genestier and Ferreira, 2017; Zhang et al., 2019). This misconception is widely considered a fact, even more than that of the hemispheric preference (Tardif et al., 2015). Teachers report having been taught about VAK/learning styles during training courses organized by their schools or the educational authorities of their governments (Lethaby and Harries, 2016; Kim and Sankey, 2017; McMahon et al., 2019). Moreover, some teachers insist they intend to continue working under the VAK perspective in their classrooms, even knowing that it is a neuromyth (Newton and Miah, 2017; Tan and Amiel, 2019).

### Neuroscience, the “Gap,” and Education

The alluring power of the images of the brain (Weisberg et al., 2008) and some surrounding related sciences, methodologies, and advances, could be seen as a new trend among researchers from multiple scientific fields. Nevertheless, the brain has been the subject of significant study, thanks to the latest technological advances, like neuroimaging. Recent research has shed light on this field (Goswami, 2006; Ansari, 2008; Geake, 2008; Gago Galvagno and Elgier, 2018). More than 30 years have passed since a United State Congressman Sylvio Conti campaigned for the end of the 20th century to be recognized as the Decade of the Brain. He encouraged organizations toward goals referring not only to investment or federal support for the topic but also to educating the public about neuroscience area (Laws, 2000).

The fields of neuroscience, cognitive neuroscience, and their possible implications in education have flourished in universities, publications, and researchers, examples being the Brain, Neuroscience Special Interest Group within the American Education Research Association or the interfaculty initiative called Mind, Brain and Behavior (MMB), launched in 1993 by Harvard University (Schwartz, 2015). As a consequence of a logical evolution (or scientific revolution), since 1999, the OECD has had a Neuroscience and Education program, as well as other institutions, such as Cambridge University, East Normal University in Shanghai (Carew and Magsamen, 2010), the University of California, and Oxford University (Cuthbert, 2015). Moreover, more recently, the Human Brain Project is the most substantial scientific project ever funded by the European Union (Kandel et al., 2013).

The combination of interest, innovation, research, and possibilities have converged in “educational neuroscience” or “neuroeducation” (Ansari et al., 2011), a crossroads for scientific research areas that can contribute to education, such as developmental psychology, cognitive neuroscience, genetics, and technology (Brookman, 2016). Due to this multidisciplinary approach, “educational neuroscience” tends to encompass research in the processes of the brain that affect learning and education (Knox, 2016). Its inherent characteristics have also promoted educational neuroscience or neuroeducation as the discipline to bridge the gap between the most theoretical part of the neurosciences and their practical contributions for education (Centre for Educational Research and Innovation and OECD, 2007; Ansari, 2008; Carew and Magsamen, 2010; Howard-Jones, 2011; Tan and Amiel, 2019).

Even though the emerging neuroeducation seemed to be received with enthusiasm, it was not without controversy and skepticism (Cuthbert, 2015) and this “gap” has always been present as a maximum representative of the distance between sciences, neurosciences, and education (Hardiman et al., 2012; Howard-Jones, 2014). Likewise, specific neuroimaging technology uses have been labeled as premature for educational research (Loftus et al., 2017). For example, Bruer (1999) has described this 30-year path almost from the beginning. He published a work directly about the hasty use of three neuroscientific findings (synaptogenesis, the critical periods, and the enriched environments) in a direct way to the educational practices (Bruer, 1999, 2016). Nevertheless, this was not a closing door—just a call to be prudent. Therefore, it is necessary to find a balance between this skepticism and the wait to apply results in the classrooms, and the simplification of neuroscience findings (Barrios-Tao, 2016).

### Educators, Neuroeducation, and Neuromyths

The advances in neuroscience and its subfields (neuroeducation, educational neuroscience) progressed adequately, at the same time as researchers explored the educational community's knowledge in these areas. It was reported that the majority of teachers and students are interested in educational neuroscience and consider it useful in their professional work (Dekker et al., 2012; Ferrero et al., 2016; Düvel et al., 2017; Bailey et al., 2018; Falquez Torres and Ocampo Alvarado, 2018; McMahon et al., 2019; Zhang et al., 2019; among others), although a minority of teachers report currently using brain-based techniques in their classrooms (Rodrigues Rato et al., 2013) or, at least, they are eager to implement those (Zhang et al., 2019). However, neuromyths can affect teachers, students, and educators when implementing neuroeducation in the school. A large selection of items is linked to the study of the neuromyths in the literature.

Neuromyths have been widely addressed from a cultural perspective. Ferrero et al. (2016) conducted an exhaustive meta-analysis to report cultural influence in the prevalence of 12 neuromyths among teachers, as some others had previously suggested (Pasquinelli, 2012; Howard-Jones, 2014; Deligiannidi and Howard-Jones, 2015; Pei et al., 2015). Ferrero's findings (Ferrero et al., 2016) showed the presence of cross-cultural differences even for neuromyths with consistent responses across ten countries (UK, Netherlands, Greece, Turkey, Peru, Argentina, Chile, other Latin American countries, China, and Spain). However, as the authors stated, similar widespread misunderstandings can be found in neuromyths in different countries (Dekker et al., 2012; Howard-Jones, 2014; Gleichgerrcht et al., 2015; Ferrero et al., 2016; Bailey et al., 2018). Since 2016, much more scientific information about neuromyths has become available, given the significant and exponential advance of neuroeducation.

Since Dekker et al. in 2012, nearly every study has tried to find the predictors of these beliefs and the protective factors, including sex, age, years of expertise, and reading “pop science” (Cooper, 1966; Brace, 1993) vs. peer-reviewed articles, with a great variety of results. A few of them have found that general knowledge of the brain is a predictor for belief in neuromyths (Dekker et al., 2012; Gleichgerrcht et al., 2015; Papadatou-Pastou et al., 2017; Varas-Genestier and Ferreira, 2017). Others have reported completing many neuroscience courses (Macdonald et al., 2017) or semesters (Düvel et al., 2017) and reading peer-reviewed scientific journals (Macdonald et al., 2017) or a broader educational background (Zhang et al., 2019) as protective items against the belief in neuromyths. In one case, being female seemed to be related to lower neuromyth scores (Dündar and Gündüz, 2016) and later, females were cataloged as more likely to agree with neuromyths (Bailey et al., 2018). However, previous research failed to find any gender difference (Dekker et al., 2012; Karakus et al., 2015).

In particular, and to the best of our knowledge, no previous study has considered exploring more than five studies and 12 neuromyths in an attempt to gather all the information available about the beliefs in neuromyths among educators. Given these mixed findings, it seems necessary to collect more scientific data around neuromyths. Furthermore, once cultural variation (Ferrero et al., 2016) and the prevalence of the neuromyths among teachers (in or pre-service) has been described, it is essential to collect as much data as possible under an updated scientific method, assembling the knowledge available to the present day.

This study aims to present a systematic methodological review to assemble the primary data in neuromyths from a time perspective, from Dekker et al. (2012) to Tovazzi et al. (2020). Thus, it is crucial to sort the data chronologically, according to the year of publication. It was our aim to lay out a timeline with the prevalence of neuromyths in recent years. Despite decades of research, no study to date, has systematically reviewed the neuromyth literature. Thus, this paper addresses neuromyths among educators as a research topic from 2012 to 2020. Neuromyths have been previously assessed only to a limited extent. Although studies have been conducted by many authors, additional analyses to explore the neuromyths recently described are required.

This manuscript contains a systematic review that is novel in terms of the methodological approach adopted and the number of items analyzed. No study to date has considered the literature on neuromyths under a rigorous review and neither is there previous research describing all the neuromyths addressed in surveys among educators.

## Methodology

### Search Strategy Design and Development

This manuscript contains a systematic review never previously conducted. No other scientific research has listed the literature on neuromyths under a rigorous review, nor, more specifically, the articles about these neuromyths disseminated among in service or preservice teachers. Two colleagues replicated the search protocol outside this research at the end of June 2020.

An exhaustive and systematic search was conducted to collect all the documents related to the topic of interest. We established four steps with an increasing degree of depth to draw up the search strategy. Thus, we set the stage to refine the search and make it tighter in every run, finding, due to this narrowing down, the most accurate and representative articles in the field of concern. In particular, the search strategy was implemented using these four words or groups of words in this order: (1) *Neuromyths*; (2) *Neuromyths and Education*; (3) *Neuromyths and Education and pre-service teachers*; and (4) *Neuromyths and Education and Teachers*. Hence, the search could be replicated introducing the terms above in the literature databases used (i.e., Web of Science, SCOPUS, EBSCOhost and the selected databases therein, ProQuest, and PubMed). To avoid publication bias, we added seven different databases in the EBSCOhost and also explored Dialnet and Google Scholar. To collect all the references and to detect the duplicates among the findings, we harnessed a reference management software to compile and compare the recorded data, specifically, *EndNote*^®^ *online version* was selected.

**Figure 1:**
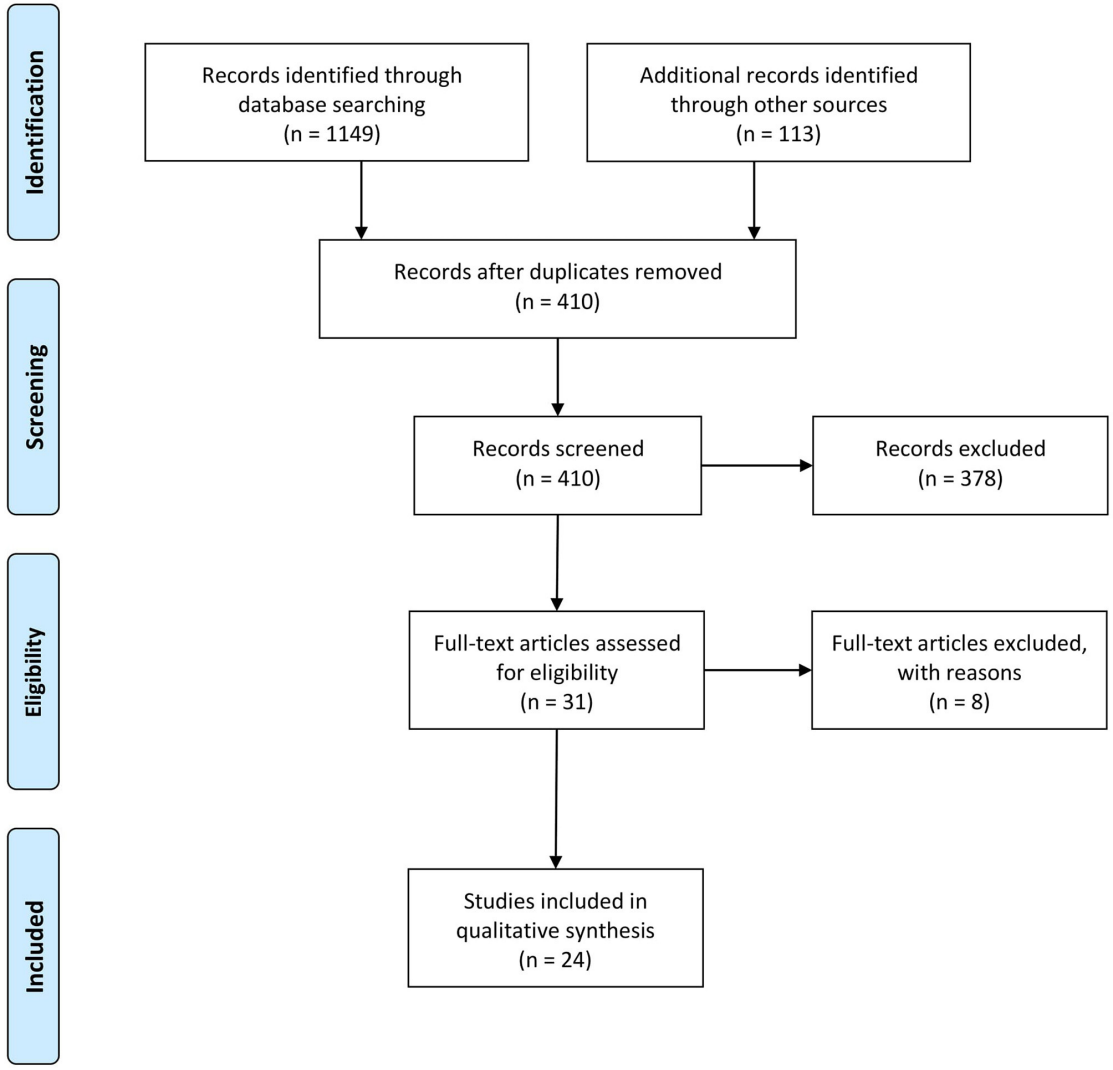
Flow diagram based on Prisma, based on Moher et al. (2009).

For a better systematization of the process, the search strategy was implemented with the template *Prisma Flow diagram* (Moher et al., 2009), which gives a summary at a glance of the work behind the data collection (Figure 1). The initial search delivered 1,262 documents matching the criteria. After removing duplicates (*n* = 852), we explored 410 records under the inclusion criteria procedure to refine the documents according to the topic and the objectives of the current research.

**Figure 2:**
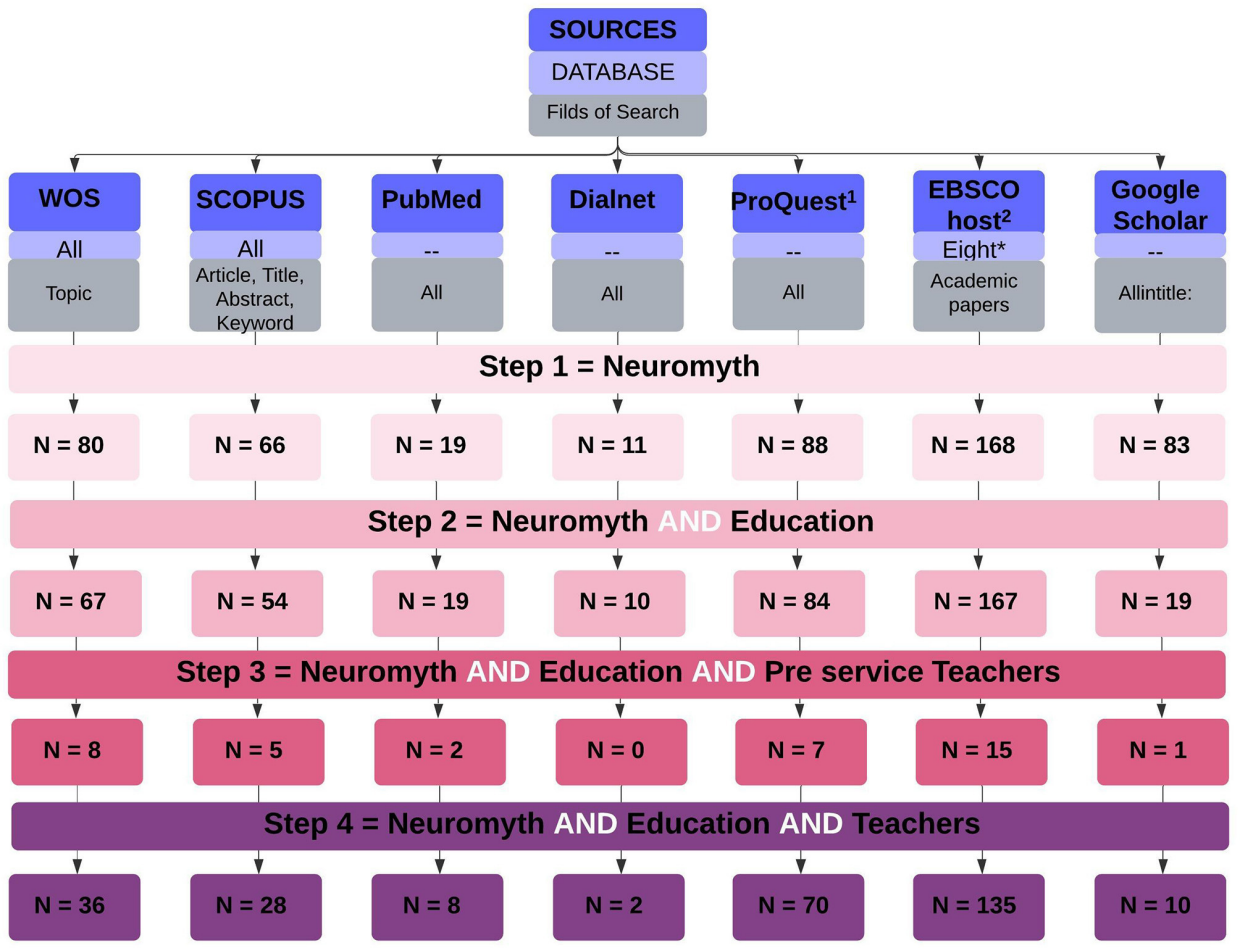
Search strategy and results. The eight* database screened in EBSCOhost are: MEDLINE; Education Source, ERIC, Psychology and Behavioral Sciences Collection; APA PsycINFO, SPORTDiscus with Full Text, Teacher Reference Center, PSICODOC.

Figure 2 is a graphical representation of the search strategy, including the keywords or group of words. A massive number of results were obtained since the search was only combined with one English Boolean data type (*and*) because there is no synonym for the word, *neuromyth*. The figure shows the three steps through which the search strategy could be redone. Stage 1 includes the word *Neuromyth* alone as search criterion. Step 2 added the word *education* to the search through the Boolean operator AND. With the same operator, Step 3 put together the first two keywords AND *preservice teachers*. Finally, not from the last search, but also the second, another branch forks out to give results for Step 4 composed of the first two terms (neuromyths and education) AND *Teachers*. With these four conditions, the whole field of interest was covered.

### Inclusion Criteria

The initial screening ended with *N* = 410 scientific articles to be reviewed. Because of this vast number, several inclusion criteria needed to be applied in the next stage of the procedure. Thus, a decision tree was made to select the articles according to the main aim and the previous standards.

**Figure 3:**
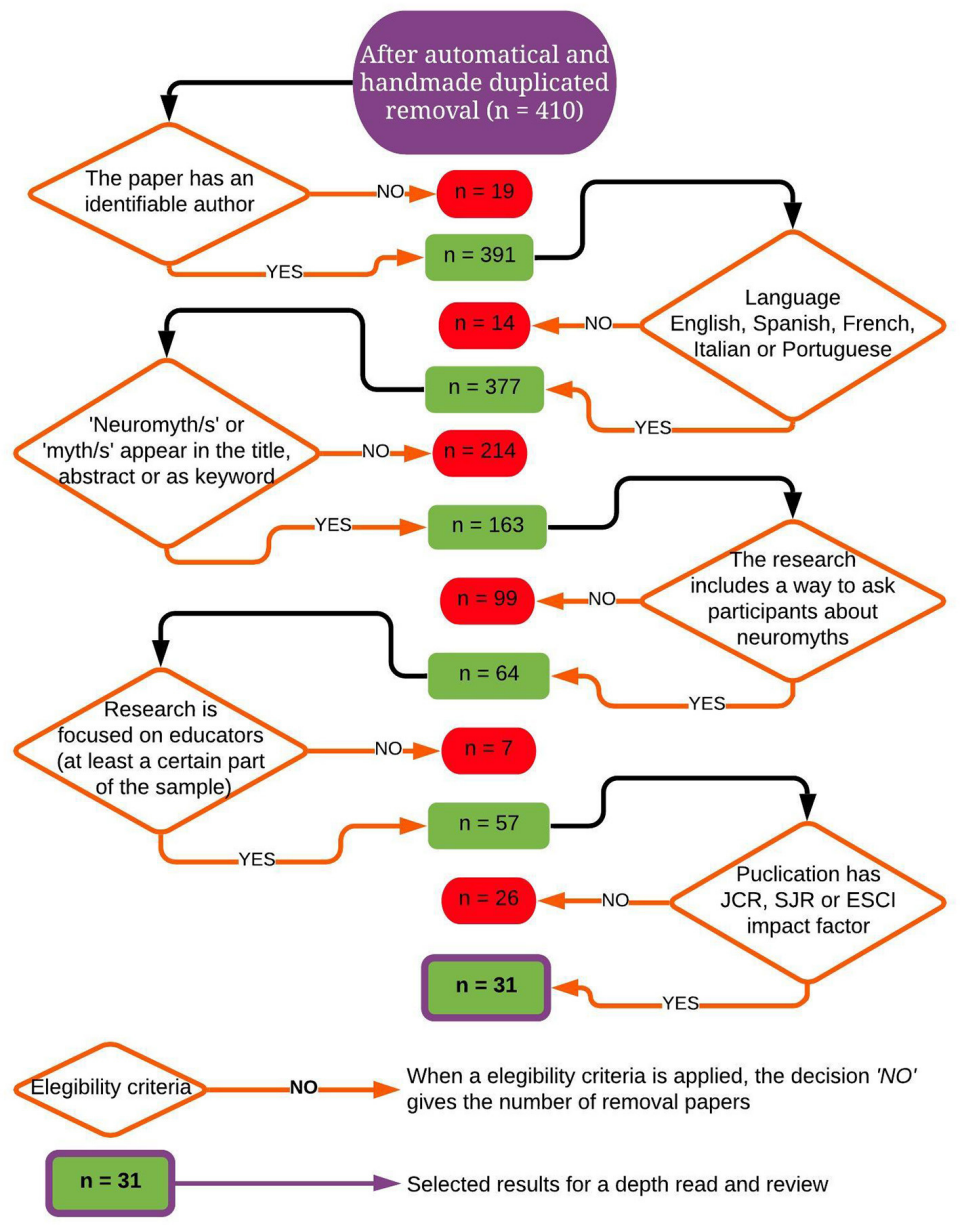
Inclusion and exclusion criteria: decision tree.

Figure 3 illustrates how, for each inclusion criterion, the decision “no” gives the number of documents removed, whereas, with a “yes” response, the article proved to be eligible to continue in the systematic review. Undoubtedly, the inclusion items are intrinsically interesting for their capacity to reduce the number of documents to be kept in the analysis. The most restrictive conditions that removed the greatest number of studies were “Neuromyth/s or myth/s appear in the title, abstract, or as keywords” (*N* = 214). Secondly, “The research includes a way to ask participants about neuromyth” left behind *N* = 98 records. When the authors of the papers were unidentifiable (*N* = 19), they were discarded. The authors of the present study are able to read in Spanish, English, French, Italian, and Portuguese, but we were forced to delete any evidence written in a different language other than those specified. The search strategy returned (*N* = 14) articles published in Japanese, Chinese, German, and a few Slavic languages which were impossible to translate properly for use in this scientific document. However, a point to bear in mind is that we cannot be sure these 14 documents fit correctly into the present systematic review because they could also have been eliminated under the light of other eligibility criteria. Documents with a sample other than teachers (in service, in training, or preservice), were removed (*N* = 7). Finally, the authors decided to apply one last restriction to the 58 remaining files. To add quality to the systematic review, we only took into account the academic studies indexed in any type of impact factor, i.e., Journal Citation Report (JCR) or the ones that could report the average number of weighted citations received in a year, as does the SJR measure. To avoid the publication bias, articles in an Emerging Source Citation (ESCI) were included in the present review.

Thus, by the end of the eligibility criteria, *N* = 31 articles of scientific research on the main topic were deemed suitable for an in-depth review. Then, to encourage an exhaustive reading attitude, a new code was designed to preserve the quality of the study. Guided by a table of contents, every one of the team members checked the 31 findings according to the closest participants, intervention, comparison, outcome (PICO) strategy possible.

Therefore, participants (P) were bounded as teachers, educators or trainers, who are related, in some way, at present or in the future time, to teaching people (any age) or to designing teaching programs. Experimental studies respond to intervention (I) questions, but this is not our case as we present a review of descriptive studies, where the research looks for frequencies in the answers (García-Perdomo, 2015). Thus, to follow PICO strategy, under our limitations, a questionnaire including at least three of the neuromyths defined by the OECD (2002) and Centre for Educational Research and Innovation and OECD (2007) and collected by the first publication that appeared in our screening, was set as intervention. The first published article in these 31 eligible documents is that of Dekker et al. (2012). We consider this the beginning of the studies in neuromyths among teachers. Nonetheless, a few studies were conducted before this one, but here we can find the objectives and methodologies defined in the research question raised in this work.

Comparisons (C) are outside the interest of this publication. Finally, the outcome (O) was closely related to the intervention (I) because we were dealing with a set of descriptive studies. The mandatory outcome was to have an overall percentage of prevalence in neuromyths (or to provide the way to calculate it from the data shown) and preferably to have rates of prevalence for each neuromyth.

When neuromyths are not specified in a study, those described in the previous papers in this review (Dekker et al., 2012) have been considered neuromyths. Consequently, in Pei et al. (2015), 9 statements among all the 38 factual assertions coincided with those collected in the previous analyzed literature. Similarly, in Dündar and Gündüz (2016), 15 items were identified before as neuromyths.

## Results

### Excluded Studies After In-depth Reading

The reading process ended by discussing the articles most suitable for exclusion from the review. In particular, to eliminate an item, it was necessary to reach more than 80% of the interjudge agreement. Under this percentage, the authors deliberated all the points of view. One paper generated disagreement among the authors because of the sample surveyed. Finally, Bailey et al. (2018) was included in the current analysis because the majority of the authors leading the research considered coaches educators who have the opportunity to influence people's education in some way. This procedure omitted seven studies (Appendix 1) from the systematic review for justifiable reasons, according to the aforementioned PICO strategy parallelism.

### Systematic Review Findings

To compile data from the concrete results under the criteria of this unique research, Table 1 shows the main findings included in the 24 articles analyzed. The references are sorted by year of publication, to obtain a panoramic view of the prevalence of neuromyths according to our main aim. The information is organized around five primary columns. The first on the left contains the principal author and year of publication of the research, then appears the sample explored and its provenance when indicated.

**Table 1:** Synthesis of the investigations about neuromyths.

**References**	**Sample**	**Methodology**			**Results**				**Main outcomes**
		**Survey design**	**Neuromyths included**	**Response type**	**Prevalence of neuromyths**	**Most Less**	**Score in GKAB**	**Neuromyths predictors or protectors**	
Dekker et al. (2012)	*N* = 242 Pr and Sc teachers from UK and NL	32 statements (OECD, 2002)	1, 2, 3, 4, 5, 6, 7, 8, 9, 10, 11, 12, 13, 14, 15	Incorrect	56.88%^*^	1, 2, 3	Average 70%	Predictor: general knowledge	- Brain knowledge has no protective effect
				Correct		11, 10, 12			
				DNK					
Rodrigues Rato et al. (2013)	*N* = 583 Portuguese PS to HS teachers	7 statements (OECD, 2002) and 1 about vitamin supplements myth	9, 16, 17, 18, 19, 20	Myth	–	17, 19, 16	–	Not found	- The interest in neurosciences does not allow to distinguish myths, especially learning
				Fact		20, 9, 18			
				DNK					
Deligiannidi and Howard-Jones (2015)	*N* = 271 Greek Pr and Sc teachers	38 factual assertions	1, 2, 3, 4, 5, 6, 7^∧^, 9, 11, 12	Agree	57.7%^*^	1, 5, 4	60.6%^*^	Not studied	- The myths believed are directly related to practice and brain-based learning programs (with no sufficient scientific basis)
				DNK		15, 12, 11			
				Disagree					
				NR					
Gleichgerrcht et al. (2015)	*N* = 3,451 Latin American ECE to HE teachers	Survey (Dekker et al., 2012) with 32 assertions	1, 2, 3, 4, 5, 6, 7, 8, 9, 10, 11, 12	Incorrect Correct DNK	50.7%	1, 5, 4 15, 12, 11	66.7%	Predictor: performance in general statements	- The significant difference across countries in neuromyths and general knowledge - Lack of scientific texts in Spanish
Karakus et al. (2015)	*N* = 278 Turkish Pr and Sc teachers	32 statements (Dekker et al., 2012)	1, 2, 3, 4, 5, 6, 7, 8, 9, 10, 11, 12, 13, 14, 15	Incorrect	53.02%	1, 5, 7	56.9%	Not found	Neuromyths are linked with commercial programs.
				Correct		15, 14, 11			
				DNK					
Pei et al. (2015)	*N* = 238 East China Pr, Sc, and HS teachers	38 items (Herculano-Houzel, 2002; Howard-Jones et al., 2009)	1, 2, 3, 4, 5, 6, 7^∧^, 9, 11, 12	Agree DNK Disagree	61%^*^	1, 5, 3 12, 7, 11	56.6%^*^	Not studied	- Cultural misunderstandings between East China and Europe - More enthusiastic about attention than emotion in reasoning
Dündar and Gündüz (2016)	*N* = 2,932 Pr and Sc preservice teachers	59 items (Herculano-Houzel, 2002; Howard-Jones et al., 2009; Dekker et al., 2012; Karakus et al., 2015)	1, 2, 3, 4, 5, 6, 7, 8, 9, 10, 11, 12, 13, 14, 15	Yes No DNK	52.72%^*^	1, 2, 5 11, 12, 8	50.1%^*^	–	- Most believed: VAK, hemispheric dominance, and the improvement of the brain by certain nutrients - Better identification of general neuromyths than educational
Ferrero et al. (2016)	*N* = 284 Spanish PS, Pr, Sc, and vocational teachers	32 statements (Dekker et al., 2012)	1, 2, 3, 4, 5, 6, 7, 8, 9, 10, 11, 12	Incorrect Correct DNK	49.1%	5, 1, 4 11, 7, 10	62.29%	- Predictors: GK and reading educational magazines - Protector: to read scientific journals	- Along the countries is spread the idea of learning style, and rich environments improve preschool children's brain - There is a substantial amount of cross-cultural variation
Hermida et al. (2016)	*N* = 204 Argentine preschool teachers	18 items (Herculano-Houzel, 2002; Howard-Jones et al., 2009)	8, 9, 11	Agree DNK Disagree	27.33%^*^	–	60.1%^*^	Not found	- Cultural conditions, media, and lack of neural basis knowledge can contribute to the misunderstanding above the neuroscience awareness
Lethaby and Harries (2016)	*N* = 128 English teachers	8 items about VAK (Howard-Jones et al., 2009; Dekker et al., 2012)	1, 2, 3, 9, 11, 21	Agree DNK Disagree	45.71%^*^	1, 2, 3 11, 16, 9	81.3%^*^	Not studied	- Beliefs influence teaching. - 88% believe in learning styles
Kim and Sankey (2017)	*N* = 1,144 Australian preservice teachers	20 statements (Dekker et al., 2012)	1, 2, 3, 5, 7	Correct Not correct	84.46%^*^	1, 3, 2 5, 7	75.6%	- Predictor: performance in general statements	- VAK and left/right myth could be learnt at school or university while *Fatty Acid Supplements* myth seems to be spread by a TV advertisement
Macdonald et al. (2017)	*N* = 598 educators	32 statements (adapted from Dekker et al., 2012)	1, 2, 3, 4, 6, 8, 9, 10, 11, 12, 14, 22, 23, 24, 25	True False	45.8%^*^	3, 4, 1	85.7%^*^	- Predictors: neuroscience courses and reading peer-review journals - Protectors: teaching higher education	- Training both neuroscience and education and the quality of the media exposures reduces the belief in neuromyths - Authors modified version revealed 1 factor with 7 neuromyths (1, 2, 6, 9, 22, 23, 24)
	Also, *N* = 234 high neuroscience exposure and *N* = 3,045 general public (groups not studied here)					14, 11, 8			
Papadatou-Pastou et al. (2017)	*N* = 573 Greek under and postgraduate students enrolled in the Department of Education	70 statements (Herculano-Houzel, 2002; Lilienfeld et al., 2011; Dekker et al., 2012) and 7 related to Special Education	1, 2, 3, 4, 5, 7, 8, 9, 10, 11, 12, 13, 14, 15, 26, 27, 28, 29, 30, 31, 32, 33	Incorrect Correct DNK	43.62%	1, 5, 4 15, 28, 12	78.94%	- Predictor: the error score for general knowledge about the brain	- No significant difference between under and postgraduates - Acquiescence bias can explain general knowledge as a predictor of neuromyths
Varas-Genestier and Ferreira (2017)	*N* = 91 Chilean Pr and Sc teachers	32 items survey (Dekker et al., 2012) translated to Spanish	1, 2, 3, 4, 5, 6, 7, 8, 9, 10, 11, 12 (analyzed neuromyths from 1 to 7)	Agree DNA	83.7%	1, 5, 2 6, 2, 4, 7 (last 3 same %)	71.4%	- Predictors: reading pop-science and informed knowledge in neuroscience	- Teachers are not capable of discriminate between science and pseudoscience due to the lack of science literacy - It is urgent to review the content in the preservice training
Bailey et al. (2018)	*N* = 545 coaches from UK and Ireland	15 statements (Dekker et al., 2012; Howard-Jones, 2014 Ferrero et al., 2016; among others)	1, 2, 3, 6, 8, 9	Incorrect Correct DNK	41.6%	1, 3, 6 8, 9, 2	56.6%	- Not found	- Coaches show a high prevalence of neuromyths but lower than teachers - The better knowledge and awareness about the brain, the more neuromyths
Falquez Torres and Ocampo Alvarado (2018)	*N* = 328 ecuatorian pregraduate students	Survey version of Dekker et al. (2012) with 32 assertions	1, 2, 3, 4, 5, 6, 7, 8, 9, 10, 11, 12	Incorrect Correct DNK	56%	1, 5, 4 12, 9, 11	54%	- Predictor: age - Protectors: interest, read neuroscience, and formal education	- The lack of neuroscience presence in the educational field facilitates the neuromyths arisen - Similar findings than in in-service previous studies samples.
Horvath et al. (2018)	*N* = 50 PS, Pr, Sc, and Tr-awarded teacher.	Survey with 15 common neuromyth (Howard-Jones et al., 2009)	1, 2, 3, 4, 5, 6, 7, 8, 9, 10, 11, 12, 14, 15, 18	Incorrect Correct DNK	47.67%^*^	1, 5, 2 15, 14, 13	Not studied	Not studied	- The scale can only be considered a series of random responses, not a kind of composite measure - The validity may be questionable
Ruhaak and Cook (2018)	*N* = 129 special education preservice teaches from the USA	Survey adapted from Dekker et al. (2012) with 10 neuromyths and 15 general statements	1, 2, 3, 4, 7, 8, 10, 11, 14, 15	Inaccurate Accurate DNK	51.24%^*^	1, 8, 2 14, 7, 15	62.5%	- Predictor: number of educational courses	- Popular press or online sources are the way to know about the brain - Inconsistent vocabulary to describe myth-based and effective practices
Škraban et al. (2018)	*N* = 131 Pr first or last year teacher education students in Ljubljana	A questionnaire with 7 neuromyths (Herculano-Houzel, 2002: Dekker et al., 2012)	1, 2, 3, 6, 8, 9, 34	Likert-type Scale (1 to 5)	53.86%^*^	1, 2, 3 34, 6, 8	Not studied	Not studied	- A significant difference between first and last year only found in neuromyth 8. - Students do not get enough scientific information, so they need specific courses about the brain
van Dijk and Lane (2018)	*N* = 169 educators from the USA	Survey adapted from previous research with 18 fact statements and 15 neuromyths	1, 2, 3, 4, 5, 6, 8, 9, 10, 11, 12, 13^∧^, 14, 35, 36	Incorrect Correct NAOD DNK	40.5%^*^	5, 3, 4 11, 13^∧^, 10	64%	- Protector: percentage of correct facts	- Learning styles, hemispheric and Brain Gym are the most widely and pervasive believed
McMahon et al. (2019)	*N* = 130 trainee teachers in England	31 items from Dekker et al. (2012).	1, 2, 3, 4, 5, 6, 7, 8, 9, 10, 11, 12, 13, 14, 15	1- to 7-point Likert-type scale	73.04%* for the 7 most prevalent ones	5, 1, 3	NS	Not studied	- More than a third of trainees confirm experiences on somehow with brain-related training - Participants tended to have more correct answers about general knowledge than about neuromyths
		The complete study is a pre-postdesign. Here, only included predata and results				–			
Sarrasin et al. (2019)	*N* = 972 teachers from PS, Pr, and Sc in Quebec	10 statements inspired by Dekker et al. (2012) and Tardif et al. (2015)	1, 2, 3, 9, 37	5-point Likert-type scale	57.8%^*^	1, 37, 2 3, 9	66.2%^*^	Not studied	- Having a postgraduate do not protect against neuromyths - Teachers (more preschoolers) report using LS or MI in the classroom - The primary sources are cognitive bias and university training
Zhang et al. (2019)	*N* = 253 headmasters from schools in China	40 statements based on Dekker et al. (2012) and Howard-Jones et al. (2009)	1, 2, 3, 4, 5, 6, 7, 8, 9, 10, 11, 12, 13, 14, 15	4-point Likert-type scale	56.81%^*^	1, 5, 2 15, 8, 12	–	- Protectors: more years of education, level education, and best score in general assertions	- In the interviews, headmasters linked their neuroscience knowledge to their own experience - The sample believes in more neuroscience statements related to the environment affection and less to the commercial products
Tovazzi et al. (2020)	*N* = 174 Italian teachers from PS, Pr, and Sc	40 questions (Deligiannidi and Howard-Jones, 2015)	1, 2, 3, 4, 5, 6, 7, 8^∧^, 9, 11, 12, 15, 38, 39	Agree Disagree DNK	56.73%^**^	1, 4, 5 39, 12, 7	43.9%^**^	- Predictor: correct answers in general assertions	- When asking teachers about real teaching scenarios is less likely to select a neuromyth as an answer
		The new questionnaire proposed is not analyzed here							

*In order of appearance: GKAB, general knowledge about the brain; Pr, primary school; Sc, secondary school; DNK, do not know; PS, preschool; HS, high school; ECE, early childhood education; HE, high education; GK, general knowledge; Tr, tertiary sector school; NAOD, nor agree or disagree; LS, learning styles; MI, multiple intelligences*.

* *The percentage has been calculated according to the given data in tables or appendixes into the article analyzed*.

** *Updated percentage with the acknowledge of the article authors (D. Basso, personal communication). ^∧^Inverse item*.

The methodology is in the third place, divided into more three columns: the survey used to collect the sample responses; the list of neuromyths included (Table 2) in the article; and the type of answers available in the survey.

**Table 2:** List of neuromyths appeared in analyzed articles.

**No**	**Neuromyths**
1	Individual learn better when they receive information in their preferred learning style (e.g., auditory, visual, kinesthetic)
2	Differences in hemispheric dominance (left brain, right brain) can help explain individual differences among learners
3	Short bouts of co-ordination exercises can improve integration of left and right hemispheric brain function
4	Exercises that rehearse co-ordination of motor-perception skills can improve literacy skills
5	Environments that are rich in stimulus improve the brains of preschool children
6	Children are less attentive after consuming sugary drinks and/or snacks
7	It has been scientifically proven that fatty acid supplements (omega-3 and omega-6) have a positive effect on academic achievement
8	There are critical periods in childhood, after which certain things can no longer be learned
9	We only use 10% of our brain
10	Children must acquire their native language before a second language is learned. If they do not do so, neither language will be fully acquired
11	Learning problems associated with developmental differences in brain function cannot be remediated by education
12	If pupils do not drink sufficient amounts of water (=6–9 glasses a day), their brains shrink
13	Regular drinking of caffeinated drinks reduces alertness
14	Extended rehearsal of mental processes can change the structure or function of some parts of the brain
15	Individual learners show preferences for the mode in which they receive information (e.g., visual, auditory, kinesthetic)
16	The left and the right brain work independently
17	There are separate types of intelligence (e.g., interpersonal, logical; with different IQs)
18	Drinking extra water (even when one is no longer thirsty) is vital for brain function
19	Learning styles should be based on multisensory pedagogies (VAK model)
20	Students should be given vitamin supplements of other medications to learn better
21	Teaching to learning styles is more important in language learning than in other types of learning
22	Children have learning styles that are dominated by particular senses
23	A common sign of dyslexia is seeing letters backwards
24	Listening to classical music increase children's reasoning ability
25	Children must be exposed to an enriched learning environment by age 3, or else learning capacities will be lost
26	The brain of children with attention-deficit hyperactivity disorder (ADHD) is over-aroused
27	IQ scores are unrelated to school performance
28	Raising children similarly leads to similarities in their adult personalities
29	Visual perceptions are accompanied by tiny emissions from the eyes
30	Human memory works like a tape recorder or video camera and accurately records the events we have experienced
31	Individuals can learn new information, like new languages, when asleep
32	Our handwriting reveals our personality
33	IQ scores almost never change over time
34	Everything that is important for brain development occurs within the first 3 years
35	Following a specific diet can help overcome certain neurological disabilities, such as ADHD, dyslexia, and autism spectrum disorders
36	Doing basic Brain Gym exercises help students to learn to read and use language better
37	Students have a predominant intelligence profile, for example, logic-mathematical, music, or interpersonal, which must be considered in teaching
38	Productions of new connections in the brain can continue into old age
39	Mental capacity is hereditary and cannot be changed by the environment or experience

*Items 13, 14, 15, and 39 have been considered “correct statements” in the papers where they had been used*.

As part of the outcome of this systematic review, the results give details about prevalence in neuromyths and general knowledge in the sample specified. The most and least prevalent neuromyths (Table 2) can easily be seen in the table. For works where predictors or protectors against neuromyths were studied, the last columns of the “Results” sections give the main findings.

Finally, the rightmost column briefly summarizes the primary conclusions accomplished through the research.

**Figure 4:**
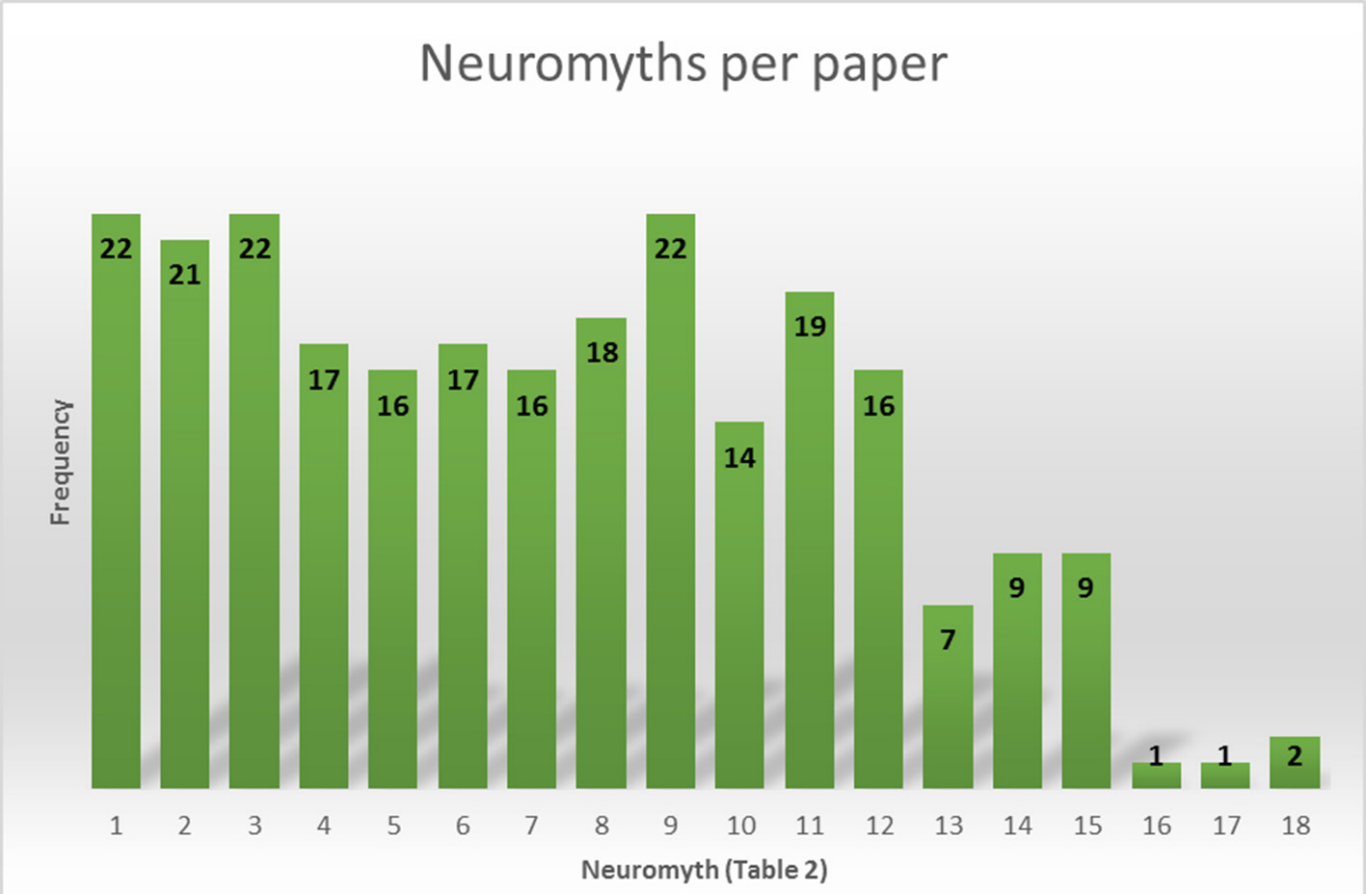
Frequency of appearance of each neuromyth in the 24 articles.

In the 24 articles explored, 39 neuromyths were surveyed in different samples. However, 23 of them appear only once. These are listed from neuromyth 19 onward (Figure 4). The three most commonly occurring neuromyths in the surveys examined are “Individuals learn better when they receive information in their preferred learning style (e.g., auditory, visual, kinaesthetic).” “Short bouts of co-ordination exercises can improve integration of left and right hemispheric brain function” and the myth that claims “We only use the 10% of our brain.”

**Figure 5:**
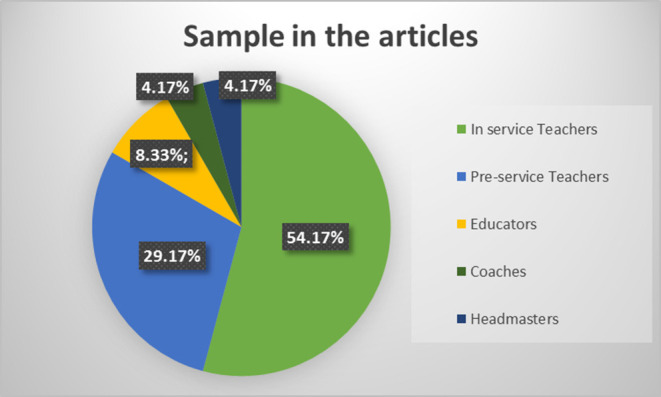
Percentage of articles by sample.

The sample scanned can be classified into five groups among the 24 papers explored. Thus, 54.17% (*N* = 13) of the documents have surveyed in-service teachers. Furthermore, as active workers, are 8.33% (*N* = 2) are unspecific educators. Coaches (*N* = 1) and head teachers (*N* = 1) account for 4.17% each. The remaining 29.17% (*N* = 7) of articles are dedicated to the preservice teachers (Figure 5).

**Figure 6:**
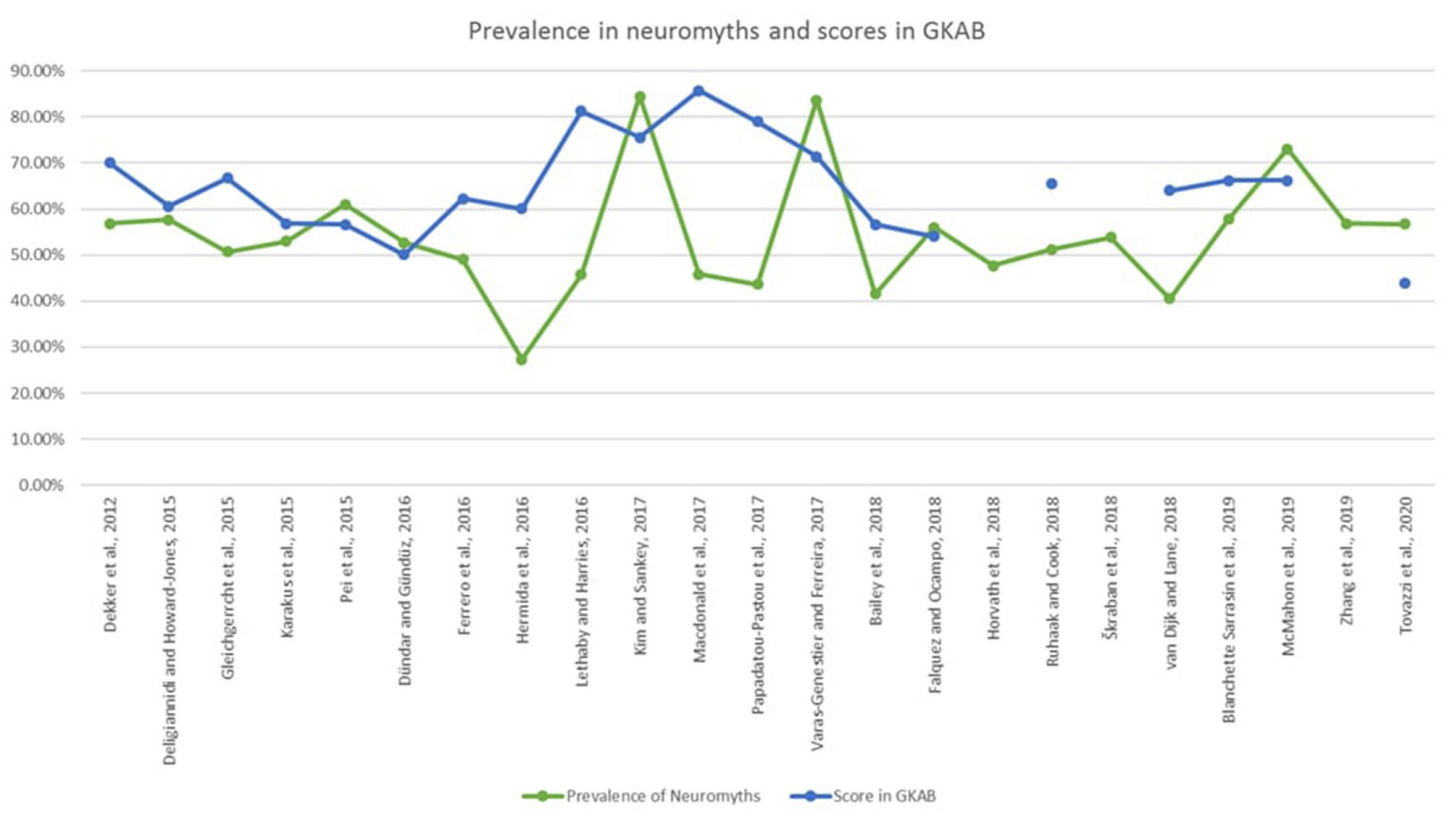
Prevalence of neuromyths and scores in GKAB from 2012 to 2020.

Finally, the findings have been presented to inform of the percentage of prevalence in neuromyths and the scores in general knowledge about the brain (GKAB), when known, across the 8 years that the current study covers (Figure 6).

The sample includes future and current teachers, with coaches, head teachers, and unspecific educators also being in the analysis. In these 24 studies, the total sample was *N* = 13,767 people involved in education, surveyed in almost 20 different countries across the world.

## Discussion

This paper has given a scientific account of the prevalence of neuromyths. Our intention was to test whether neuromyths are still present in the teachers, and with them, in education. The evidence demonstrates the existence of a large number of publications related to neuromyths among educators. It is important to note that when a false belief is taken for granted, teachers feel confident about their knowledge (Kim and Sankey, 2017). Myths can be more believable than non-myths, and people seem to be more willing to transmit them (Mercier et al., 2018). Together, the present findings allow us to recognize the most and least widely believed neuromyths. The main reason to systematize the process was also to find an evolution of the neuromyths through the years.

A total of 91.3% (*N* = 21 documents) of the research highlights as one of the most prevalent neuromyths “Individual learn better when they receive information in their preferred learning style (e.g., auditory, visual, kinaesthetic).” This is mainly the most common belief among teachers, educators, and students. Consequently, teachers around the globe believe in the effectiveness of learning styles while teaching. Teachers use this in their practice influenced by their beliefs (Willingham et al., 2015; Lethaby and Harries, 2016) of understanding the variety of learning styles (Rodrigues Rato et al., 2013). This also occurs in higher education, and what is even more dramatic is that when a professor indicated there was no empirical evidence for VAK learning (Rohrer and Pashler, 2012; Grospietsch and Mayer, 2018), 46% claimed they would find benefits from using it in class (Newton and Miah, 2017). However, previous reports confirm that there is no relation between a student's self-evaluation about their preferred learning style and the style the teacher attributes to them (Papadatou-Pastou et al., 2018), with <50% of agreement between student self-report and a learning style questionnaire (Krätzig and Arbuthnott, 2006). The fact is that we do not learn using just one sense, and VAK learning does not explain how the brain learns (Geake, 2008; Dekker et al., 2012). Using this as a theory or a valid explanation is just a teaching heuristic based on observations (Schwartz, 2015), an over-simplification (Purdy and Morrison, 2009), and a more than questionable practice (Bailey et al., 2018). In line with previous studies, this neuromyth still appears in training, education degrees, universities, or books (Gleichgerrcht et al., 2015; Lethaby and Harries, 2016; Kim and Sankey, 2017; Grospietsch and Mayer, 2018; McMahon et al., 2019; Tan and Amiel, 2019), sometimes as a general educational trend (Papadatou-Pastou et al., 2018).

More than half of the studies (58.3%) describe the sample's agreement with the demonstrated 3-year myth (Bruer, 1999) typically confused with the rapid synapsis growth happening over the first 3 years (van Dijk and Lane, 2018) and the plasticity of the brain (Im et al., 2018). A hypothesis for this scientific mistake is the confusion between the presence of a stimulus and the child's interaction with it as a cause for brain development (Pasquinelli, 2012; van Dijk and Lane, 2018). This high percentage suggests that educators neglect brain maturation and its intrasubject differences as evidence against this myth (Pasquinelli, 2012). Although it is true that considerable differences in the environment (Goswami, 2004) and an extreme sensory deprivation could be fatal for the brain, enriched environments do not necessarily improve brain development (Goswami, 2005; Centre for Educational Research and Innovation and OECD, 2007).

The last of the three most commonly believed neuromyths, with 41.7% of references among the studies, is “Differences in hemispheric dominance (left brain, right brain) can help explain individual differences among learners.” This affirmation is linked to the idea that emotional processing occurs in the right hemisphere, but grammar is in charge of the left one (Dündar and Gündüz, 2016). Undoubtedly, this is nothing other than an overgeneralization of hemispheric specialization (Ferrari and McBride, 2011). Teachers in secondary education have been shown to believe this more than teachers in primary schools (Tardif et al., 2015).

One limitation of our work is that it is not possible to give a list of the less-prevalent neuromyths due to the diversity of items (*N* = 39) compiled in these lines. The most widely explored neuromyths (Figure 3) are those from Dekker et al. (2012), so we can consider that list as the primary one in the field of study. However, the last three neuromyths described by those authors have become less common over time. Several authors (Gleichgerrcht et al., 2015; Ferrero et al., 2016; Falquez Torres and Ocampo Alvarado, 2018) have considered these as general knowledge about the brain. The definition of myth implies “a commonly believed but false idea” (Oxford University Press, 2010). Accordingly, the items about the impact of caffeinated drinks on alertness, how the mental process can change some parts of the brain and learning modality preferences in students (Table 2), are recognized as factual assertions by the abovementioned authors. Therefore, a neuromyth has to be false in itself. This is important to correctly interpret the results since this can compromise the scientific findings in previous and future research.

The academic issues above are not the only controversial methodological points in neuromyths research over the years. Some neuromyths have been formulated for specific investigations with more or less scientific rigorousness. Thus, we can find neuromyths regarding dyslexia (Macdonald et al., 2017), special education (Papadatou-Pastou et al., 2017), or neurodevelopmental disorders (van Dijk and Lane, 2018) among others (Düvel et al., 2017).

Quantitative analysis is another item of discussion. Although, indeed, neuromyth research has no complex statistical process (prevalence, ANOVA and few factorial analyses), the discordance is more than evident. The most optimistic data show a single factor with seven neuromyths in it (Macdonald et al., 2017) from a modified version of Dekker et al. (2012). Others have shown worse results, warning about the inexistence of such a factor, which questions the reliability of the measurements in the previous research (Horvath et al., 2018). For this reason, emerging research is looking for better options to study neuromyths through conceptual change (Grospietsch and Mayer, 2018) or the implication of the sociocognitive bias (van Elk, 2019). It can be delimited by putting teachers in a real and contextualized educative situation (Tovazzi et al., 2020) to discover how likely they are to use false information to decide about their professional practices.

Cultural differences are a common form of evidence in the literature analyzed (Deligiannidi and Howard-Jones, 2015; Pei et al., 2015; Ferrero et al., 2016), which is consistent with the findings of Ferrero et al. (2016). This basic outcome is consistent with research identifying religious reasons (Deligiannidi and Howard-Jones, 2015; Papadatou-Pastou et al., 2017), something being lost in translation when the sample needs to be asked in their mother tongue (Gleichgerrcht et al., 2015; Pei et al., 2015; Ferrero et al., 2016) or a sincere belief in how genes or the environment affect education (Gleichgerrcht et al., 2015; Pei et al., 2015) as a frequent cultural disparity.

The myth of “environments that are rich in stimulus improve the brains of preschool children” is particularly prevalent in most countries (Ferrero et al., 2016). It appears every year since 2015 as one of the three most pervasive neuromyth in publications. However, this neuromyth varies in prevalence across countries. Dekker et al. (2012) found a 95% of false believes in the UK vs. 56% for the same assertion in the Netherlands. Likewise, the multilingualism myth is more prevalent in designated geographical areas where this is a controversial social issue, like Turkey, Latin America, or the USA (Gleichgerrcht et al., 2015; Karakus et al., 2015; van Dijk and Lane, 2018). Considerable attention must be paid to cultural differences as they may alter the percentages of prevalence not only in neuromyths but also in general assertions about the brain (Gleichgerrcht et al., 2015).

Beyond sociocultural differences, it is not clear whether there exist protectors or predictors of the belief in neuromyths. The findings are contradictory, for example, about gender. While Dündar and Gündüz (2016) found males more likely to believe in neuromyths, Ferrero et al. (2016) pointed to women. A substantial disagreement is evident, as several authors found no significant differences by gender or other characteristics of their samples (Rodrigues Rato et al., 2013; Karakus et al., 2015; Hermida et al., 2016). Unfortunately, it was not possible to investigate the source of this diversity of results due to the heterogeneity of the methodologies and materials represented in the articles analyzed.

Despite a lack of large effects, the literature evaluated has shown that the higher the score in general knowledge about the brain, the fewer are the neuromyths correctly identified (Dekker et al., 2012; Gleichgerrcht et al., 2015; Ferrero et al., 2016; Canbulat and Kiriktas, 2017; Kim and Sankey, 2017; Papadatou-Pastou et al., 2017; Varas-Genestier and Ferreira, 2017; van Dijk and Lane, 2018; Tovazzi et al., 2020). Nevertheless, studies have also revealed opposing data (Dekker et al., 2012; van Dijk and Lane, 2018; Zhang et al., 2019). Moreover, the neuroscience literacy of the sample has been shown to be an ineffective protective factor against misconceptions (Gleichgerrcht et al., 2015). Once again, this apparent lack of correlation can justify the need for new scientific protocols in the field of neuroeducation.

Other issues studied revealed low predictive power in items such as reading pop science or educational magazines (Ferrero et al., 2016; Varas-Genestier and Ferreira, 2017), the self-informed knowledge about the brain given by the sample (Varas-Genestier and Ferreira, 2017), being 1st- or 4th-year student of science (Dündar and Gündüz, 2016) or being older (Falquez Torres and Ocampo Alvarado, 2018).

In the list of factors that can protect against neuromyths, teaching in higher education is related to being more likely to believe fewer neuromyths (Macdonald et al., 2017), which is consistent with the worse results of preschool teachers when these have been compared (Sarrasin et al., 2019). Other protectors include reading journals (Dündar and Gündüz, 2016), specifically, scientific or peer-reviewed journals (Ferrero et al., 2016; Macdonald et al., 2017; Falquez Torres and Ocampo Alvarado, 2018). Additionally, education level or having more years of formal education (Fuentes and Risso, 2015; Falquez Torres and Ocampo Alvarado, 2018; Zhang et al., 2019) and interest (Dekker et al., 2012; Falquez Torres and Ocampo Alvarado, 2018) and courses in neuroscience (Macdonald et al., 2017; Ruhaak and Cook, 2018) are related to better scores on neuromyths. This suffers the same limitations associated with the method and materials used. The absence of a corpus of specific literature in each type of sample analyzed here is a potential limitation in the search for protectors or predictors for the neuromyths.

Despite the aforementioned protectors, it is remarkable that despite education or neuroscience classes, training or the effort to present evidence against the neuromyths, these persist and are widespread (Willingham et al., 2015; Macdonald et al., 2017; Im et al., 2018; Grospietsch and Mayer, 2019; Rogers and Cheung, 2020). University students may still persist in their beliefs after a course on educational psychology or neuroscience (Macdonald et al., 2017; Im et al., 2018) or despite the empirical evidence (Petitto and Dunbar, 2004). It may be that the attempts to debunk neuromyths generate the strongest beliefs (Newton and Miah, 2017). It is not possible to improve the understanding of the brain when educators and student have to fight against a lack of scientific knowledge (Hermida et al., 2016; Varas-Genestier and Ferreira, 2017; Ruhaak and Cook, 2018; Škraban et al., 2018; Grospietsch and Mayer, 2019). Sometimes this ignorance is due to a lack of access to the neuroscientific literature because the language in question (Gleichgerrcht et al., 2015; Ferrero et al., 2016). However, other times, people point to formal education at any level—from schoolteachers to university—as the source of their false beliefs (Lethaby and Harries, 2016; Kim and Sankey, 2017; van Dijk and Lane, 2018).

Admittedly, in some cases, the mistakes respond to the great distance between scientists and educators discussed above. This bridge can be built from other disciplines, such as educational and cognitive psychology (Mason, 2009), or by paying attention to the new field of neuroeducation (Tokuhama-Espinosa, 2010). However, the foremost solution is to improve the communication between teachers and neuroscience (Dekker et al., 2012) in a standard, understandable language (Papadatou-Pastou et al., 2017; Ruhaak and Cook, 2018).

Teachers (aka educators) currently working, and students, still have difficulties to identify misunderstandings related to the brain or the neurosciences (Tovazzi et al., 2020). Some of them are more likely to fail to recognize educational neuromyths than general misconceptions related to the brain (Dündar and Gündüz, 2016; McMahon et al., 2019). The difficulty to distinguish the scientific facts from the myths is a dangerous reality in schools, colleges, and universities (Rodrigues Rato et al., 2013). Educators are under the seductive allure of neuroscience (Weisberg et al., 2008), which is more powerful when explanations are accompanied by images of the brain (Im et al., 2017) even though this reasoning is erroneous or irrelevant (McCabe and Castel, 2008). Thus, pseudoscience, media, and commercials or advertisements can have a negative impact on teachers (Gleichgerrcht et al., 2015; Karakus et al., 2015; Hermida et al., 2016; Kim and Sankey, 2017; Macdonald et al., 2017) occupying space in the classrooms as if they were proven theories (Lethaby and Harries, 2016; Sarrasin et al., 2019).

Specifically, educators claim that the Internet (Rodrigues Rato et al., 2013; Ferrero et al., 2016; Ruhaak and Cook, 2018), science or popular education magazines (Ferrero et al., 2016; Bailey et al., 2018; Tovazzi et al., 2020), and social networking (Bailey et al., 2018) are reliable sources of information. The apomediation concept described for Medicine 2.0 (Eysenbach, 2008) refers to substituting the traditional experts and authorities in health care as intermediaries and obtain information by oneself. In light of the data presented here, apomediation has arguably reached the educational field.

An appropriate starting point to fight against neuromyths is in the university curricula. Educators and education itself would benefit from accurate scientific content about the brain (Fuentes and Risso, 2015; Pei et al., 2015; Papadatou-Pastou et al., 2017). It would be a considerable achievement to transfer knowledge from the neuroscience to the teachers training (Deligiannidi and Howard-Jones, 2015; Karakus et al., 2015; Ferrero et al., 2016; Varas-Genestier and Ferreira, 2017; Falquez Torres and Ocampo Alvarado, 2018; Škraban et al., 2018). In addition, this is an opportunity to commit to lifelong learning with courses for in-service teachers because no significant differences have been found between students and professionals (Falquez Torres and Ocampo Alvarado, 2018) even in the case of award-winning teachers (Horvath et al., 2018).

## Conclusions

Taking an in-depth look into recent years of research in neuromyths, we can affirm they exist and persist among students, teachers, coaches, educators, and head teachers. We would have like to find a decreasing number of publications about the prevalence of the misconceptions related to brain into the education systems, but this is unfortunately not the case.

The distance between neuroscience and education is still too great. We have found reasons for the lack of knowledge among educators about science and the brain. Additionally, they have difficulties in accessing to the latest findings due to the absence of scientific literature in their mother tongue or the weakness of science communication.

Despite the limitations, we shed valuable light on the opportunities and the challenges of neuroeducation to enhance the scientific method in education systems. First, we have to solve the methodological drawbacks in the research on neuromyths. Future research is needed to define rigorous guidelines to identify a new neuromyth or debunk another. Undoubtedly, this guideline has to be built on the basis of academic criteria and science. This work has revealed the urgency of finding new ways to survey teachers about their perceptions, their cognitive bias, and their sincere beliefs. Moreover, access to knowledge could avoid widening the gap between neuroscience and education as a result of cultural conditions (Hermida et al., 2016).

Furthermore, specific protocols for research and systematic reviews in education or neuroeducation as an independent field of knowledge will act as a tool to reveal the importance of these kinds of approaches. The prospect of being able to use standard measures to compare data properly could prove an important area for future research.

The scientific curricula for undergraduate and postgraduate students should be revised and updated. Neuroscience has to find the language and the space to provide continuous investigation where teachers should be the leading players. In addition, teachers and students prefer to search by themselves for information, bypassing an expert intermediary. In future research, data about the sources that students use to get information would be more than welcome. Thereby, we could avoid the phenomenon of apomediation in education, finding appropriate expert mediators who seem trustworthy, skilled, and scientifically accurate, to enhance the practice of professional future teachers. Research into solving this problem is already underway. To further our research, we intend to explore the current academic curricula for trainee teachers in different countries. The design and development of neuroeducation for university studies will be a challenge.

Research has to move into the classroom at every level. In particular, it is essential to count on university teachers who are active researchers. In the same way, professionals at all teaching levels, from preschool to high school, need a better, more fluid relation with the ongoing science findings. In this case, the bridge will be made by a firm and close engagement between schools and universities. Fresh data under a standard method is needed, which gives researchers the possibility to compare, replicate and, most importantly, to advance in the knowledge to fight against the misconceptions among educators and into the education itself.

## Data Availability Statement

The original contributions presented in the study are included in the article/Supplementary Materials, further inquiries can be directed to the corresponding author/s.

## Author Contributions

All authors were involved in the design of the systematic review and in the process of evaluating the after read excluded articles. AB-O was in charge of the eligibility criteria selection. MT-M was responsible for the summarizing process and the elaboration of tables and figures. Overall, the three authors wrote the article and carried out the systematic search. Finally, SG-V reviewed and improved the text.

## Conflict of Interest

The authors declare that the research was conducted in the absence of any commercial or financial relationships that could be construed as a potential conflict of interest.
