# Supplementary material for: The Persistence of Neuromyths in the Educational Settings: A Systematic Review
Source: Front Psychol. 2021 Jan 12;11:591923. doi: 10.3389/fpsyg.2020.591923 (PMC7835631; doi:10.3389/fpsyg.2020.591923)
Supplement: Supplementary file 1 [file Table_1.pdf]

Appendix 1

Exclusion criteria

Excluded articles after reading (PICO strategy)				
Paper	Participants	Intervention / Methodological issues	Outcome	Observations
1	YES	NO	YES	Only three neuromyth analysed through new items.<br>Learning styles consider only Visual and Auditory styles.
2	YES	NO	NO	It is a pre-post study related to neuromyths in music with new items only for this approach.
3	YES	NO	NO	It is a pre-post study of neuromyths after a psychology course.<br>Only false assertions believed by the 50% of the sample or more, were considered neuromyths.
4	YES	NO	NO	Unknow neuromyths analysed.<br>The paper is focused on conceptual change, not in the prevalence of neuromyths
5	YES	NO	NO	Percentage by neuromyth not given.<br>The purpose is to know if the neuromyth will be transmitted by teachers, but not the prevalence in neuromyths
6	YES	NO	NO	The paper about Learning Styles only and the discrepancy between students and teachers regarding the topic mentioned
7	YES	NO	NO	The paper aim is misconceptions and how teachers could be transmitting these mistakes into pedagogical practice
